# Detection of IgG antibodies against the receptor binding domain of the spike protein and nucleocapsid of SARS-CoV-2 at university students from Southern Mexico: a cross-sectional study

**DOI:** 10.1186/s12879-024-09435-5

**Published:** 2024-06-12

**Authors:** Jesús Adolfo Bailón-Cuenca, Karen Cortés-Sarabia, José Legorreta-Soberanis, Víctor Manuel Alvarado-Castro, Ulises Juárez-Baltazar, Belén Madeline Sánchez-Gervacio, Amalia Vences-Velázquez, Marco Antonio Leyva-Vázquez, Oscar Del Moral-Hernández, Berenice Illades-Aguiar

**Affiliations:** 1 Laboratorio de Inmunobiología y Diagnóstico Molecular, Facultad de Ciencias Químico Biológicas, Universidad Autónoma de Guerrero, Chilpancingo de los Bravo, Guerrero, México; 2 Laboratorio de Biomedicina Molecular, Facultad de Ciencias Químico Biológicas, Universidad Autónoma de Guerrero, Chilpancingo de los Bravo, Guerrero, México; 3 Centro de Investigación de Enfermedades Tropicales, Universidad Autónoma de Guerrero, Acapulco, Guerrero México; 4 Laboratorio de Virología, Facultad de Ciencias Químico Biológicas, Universidad Autónoma de Guerrero. Chilpancingo de los Bravo, Guerrero, México

**Keywords:** Seroprevalence, SARS-CoV-2, COVID-19, IgG, University students, Spike

## Abstract

**Background:**

Natural infection and vaccination against SARS-CoV-2 is associated with the development of immunity against the structural proteins of the virus. Specifically, the two most immunogenic are the S (spike) and N (nucleocapsid) proteins. Seroprevalence studies performed in university students provide information to estimate the number of infected patients (symptomatic or asymptomatic) and generate knowledge about the viral spread, vaccine efficacy, and epidemiological control. Which, the aim of this study was to evaluate IgG antibodies against the S and N proteins of SARS-CoV-2 at university students from Southern Mexico.

**Methods:**

A total of 1418 serum samples were collected from eighteen work centers of the Autonomous University of Guerrero. Antibodies were detected by Indirect ELISA using as antigen peptides derived from the S and N proteins.

**Results:**

We reported a total seroprevalence of 39.9% anti-S/N (positive to both antigens), 14.1% anti-S and 0.5% anti-N. The highest seroprevalence was reported in the work centers from Costa Grande, Acapulco and Centro. Seroprevalence was associated with age, COVID-19, contact with infected patients, and vaccination.

**Conclusion:**

University students could play an essential role in disseminating SARS-CoV-2. We reported a seroprevalence of 54.5% against the S and N proteins, which could be due to the high population rate and cultural resistance to safety measures against COVID-19 in the different regions of the state.

**Supplementary Information:**

The online version contains supplementary material available at 10.1186/s12879-024-09435-5.

## Introduction

Coronavirus disease (COVID-19) is associated with the infection by the SARS-CoV-2. Initially, it was detected as an atypical viral pneumonia in Wuhan, China, in December of 2019 [1, 2]. The clinical symptoms of COVID-19 include cough, fever, myalgia, fatigue, diarrhea, and nauseas, among others [3, 4], it can also could evolve into severe pneumonia, dyspnea, multiple organ dysfunction, and the death of the patient [5]. SARS-CoV-2 is a positive-sense single-stranded RNA virus (+ ssRNA) of ~ 30 kb [6] that contains fourteen ORF (open reading frames) that encodes for nine accessory proteins, sixteen non-structural proteins (nsp 1–16), and four structural proteins: spike (S), envelope (E), membrane (M) and Nucleocapsid (N) [7, 8]. The S protein has 1273 amino acids length and a molecular weight of 180 kDa, contains two subunits (S1 and S2), and its primary function is the binding to the cellular receptor Angiotensin-converting enzyme 2 (ACE2) by the Receptor Binding Domain (RBD) [9]. Due to this function, the S protein is considered the main target during the design and development of vaccines. Meanwhile, the N protein has a 419 amino acids length and a molecular weight of around 45 kDa, is associated with viral replication and transcription, and maintains the ribonucleoprotein complex (RNP) [10]. Both antigens have been identified as highly immunogenic [11, 12], and their role in the host immune response has been tested by searching for IgG antibodies [13].

During the early stages of the disease, it has been proved that detecting antibodies against the N protein is more sensitive than anti-S [11]. The presence of IgM and IgG antibodies has been used to diagnose and confirm late cases of COVID-19, or to evaluate collective immunity [14], It provides evidence about natural infection due to virus spread or vaccine’s efficacy around the world, to establish new health policies and for epidemiological control [15]. The prevalence of IgG antibodies varies according to the elapsed time, country, and analyzed population [16]; during 2020, the reported prevalence was around 3.8–13.1% [17–20]. One study in India reported a seroprevalence of 20.7% and 69.2% during the first and second wave of COVID-19, respectively [21]. In Mexico, a seroprevalence of 21.3% was reported in asymptomatic patients [22], whereas in serum samples collected from February to December 2020, the seroprevalence was 33.5% [23].

In University students, a prevalence of around 4.0–4.7% was reported during 2020 in the United States [24–26], which increased to 46.8% in 2021, according to a study performed in the University of North Carolina [27]. Whereas in Brazil, the seroprevalence was 22.5% [28], in Mexico, one study reported a seroprevalence of 18.7% and 26.7%, in kids and teenagers, respectively [29]. Which it is necessary to perform studies focused on evaluating the seroprevalence of IgG antibodies against the S and N proteins of SARS-CoV-2 in university students to understand the immunization degree, provide information about the dynamic of the disease and associated factors, which was the main objective of this study.

## Materials and methods

### Study design

We developed a cross-sectional study that included a total of 1418 participants who were randomly selected from eighteen educative centers of the Universidad Autónoma de Guerrero located in six regions of the state of Guerrero (Supplementary material 1). The state of Guerrero has a territorial extension of 63,794 km^2^ and is located in the Southern region of Mexico (southern coordinates over the Pacific Ocean between the 16°18’57.60"N to 18°53’16.08"N of north latitude and 102°11’02.40"W 98°00’26.28"W of west longitude) (Supplementary material 2).

### Sample collection and survey

Sample collection was performed in each educational center from November 2022 until May 2023. A blood sample was collected in a tube without anticoagulant by venipuncture; later, the tube was centrifuged at 3500 rpm for 10 min. The serum sample was placed into a new tube and stored at -20 °C. Clinical and health data was collected by a survey that included personal (name, age, and contact number) and health data (COVID-19, number of past infections, symptoms, hospitalization, previous vaccination, number of doses, and chronic diseases).

### Indirect ELISA for IgG anti-S and anti-N

For antibody detection against the Spike protein of SARS-CoV-2, we used as antigen five peptides located in the RBD S1 domain of S protein and one peptide in the N protein; both antigens were synthesized as multi-antigenic peptides of eight ramifications (MAP8) as previously reported by our workgroup [30]. A clinical sensitivity of 92% and a specificity of 96% was obtained for both antigens, which is also depicted by the corresponding receiver operating characteristic (ROC) curve obtaining an area under the curve of 0.944. The ROC curve was calculated with 95% confidence intervals (0.89 to 0.99).

First, microtiter plates (Sigma-Aldrich) were coated with 100 µL/well of each antigen to a final concentration of 0.1 µg/mL in a coating buffer (50 mM Na_2_CO_3_/NaCO_3_H, pH 9.6). The plates were incubated for 1 h at 37 ºC and then blocked for 30 min at 37 ºC with 200 µL/well of 5% skimmed milk diluted in phosphate-buffered saline (PBS)-Tween 20 (0.05%). For the primary antibody, 100 µL/well of serum samples (1:50 dilution) were incubated by a duplicate for 45 min at 37 ºC. Later, 100 µL/well of mouse monoclonal anti-human IgG (Sigma-Aldrich; dilution 1:1500) was added for 45 min and incubated at 37 °C. After every step, the plates were washed thrice with 200 µL/well of PBS-Tween 20 0.05% for 5 min. The enzymatic reaction was developed using o-phenylenediamine dihydrochloride (Sigma-Aldrich) and stopped with 2 N H_2_SO_4_. The optical density (OD) was measured at 492 nm using a microplate reader. Samples with an OD > 0.250 were considered positive for both antigens.

### Statistical analysis

The analysis relied on the statistical package CIETmap 2.1, a Windows interface for the R programming language [31]. We performed a univariate analysis and obtained descriptive results and simple frequencies. We examined descriptive data as frequency of each analyzed variable and measures of central tendency. The association between the presence of antibodies against the S and N proteins and analyzed variables was determined by bivariate and multivariate analysis, using the Mantel-Haenszel procedure [32]. Multivariate analysis began with a saturated model that included all the statistically significant variables associated with the outcome in bivariate analysis, removing the less significant associations one by one until only associated variables with the outcome at the 95% confidence level remained. We reported associations as odds ratios (OR) with 95% confidence intervals (95% CI) were calculated by the Miettinen method [33], and a significant P value < 0.05 was considered statistically significant. The territorial map of the state of Guerrero was performed in the software ArcMap V.10.8, used as a template for online maps. Later, by using Google Earth, we added the UTM coordinates of each center of the Universidad Autónoma de Guerrero, in which sampling was performed.

## Results

We surveyed 1418 college students with a mean age of 20.5 years (range 18–29). 67% were women, of which 2.3% were pregnant. The 45.3% had COVID-19, and the main symptoms were fever (78.9%), cough (67%), and sore throat (66.9%). Also, 98.7% received the complete vaccination scheme (two doses for almost all the included vaccines with the exception of CanSino and Johnson & Johnson) and 77.1% had a heterologous dose (Table 1). The vaccines applied in the second and booster doses are shown in supplementary material 3.

**Table 1:** Symptoms, comorbidities, COVID-19 infection and vaccination status

Variable	Categories	% (*n*)
**Gender**	Male Female	33 (466/1418) 67 (952/1418)
**Symptoms**	Fever	78.9 (498/631)
	Cough	67 (423/631)
	Sore throat	66.9 (422/631)
**Comorbidities**	Obesity	23.1 (327/1418)
	Asthma	6.3 (90/1418)
	Hypertension	1 (14/1418)
	Diabetes	0.6 (9/1418)
	Cancer	0.3 (4/1418)
**Reported direct contact with a COVID-19 patient during the pandemic**	Yes	62.6 (887/1418)
**Reported COVID-19 at least one time**	Yes	45.3 (642/1418)
**Complete vaccination scheme against COVID-19**	Yes	98.7 (1399/1418)
**Had a boost dose**	Yes	67.7 (951/1405)
**Vaccine**	AstraZeneca	26.7 (375/1405)
	SinoVac	26.0 (366/1405)
	CanSino	23.2 (326/1405)
	Pfizer/BioNTech	22.3 (314/1405)
	Others*	1.8 (24/1405)
**Boost**	Homologous	22.9 (322/1405)
	Heterologous	77.1 (1083/1405)

* Sputnik V, Moderna and Johnson & Johnson

We found a global seroprevalence of 14.1% for anti-S, 0.5% for anti-N, and 39.9% for both antigens (anti-S and anti-N). The highest seroprevalence for anti-S was the region of Costa Chica (18.7%) and Tierra Caliente (16.9%), while, for anti-S and anti-N was Costa Grande (57.7%) and Acapulco (51.8%), significant differences were observed between the reported prevalence among regions (Table 2).

**Table 2:** Seroprevalence of antibodies against proteins S and N in the six regions studied

Results	Global	Regions						*P* value
		Costa Grande	Acapulco	Centro	Costa Chica	Tierra Caliente	Norte	
**Negative**	**45.5%** (646/1418)	**28.8%** (15/52)	**33.4%** (185/554)	**50.8%** (187/368)	**55.4%** (198/177)	**59.3%** (77/130)	**61.3%** (84/137)	< 0.001
**IgG anti-S**	**14.1%** (200/1418)	**13.5%** (7/52)	**14.6%** (81/554)	**11.1%** (41/368)	**18.7%** (33/177)	**16.9%** (22/130)	**11.7%** (16/137)	< 0.001
**IgG anti-N**	**0.5%** (6/1418)	**0%** (0/52)	**0.2%** (1/554)	**0.9%** (3/368)	**0%** (0/177)	**1.5%** (2/130)	**0%** (0/137)	< 0.001
**IgG anti-S and IgG anti-N**	**39.9%** (566/1418)	**57.7%** (30/52)	**51.8%** (287/554)	**37.2%** (137/368)	**25.9%** (46/177)	**22.3%** (29/130)	**27.0%** (37/137)	< 0.001

Subsequently, we performed a bivariate test in which we showed that the presence of IgG anti-S was associated with age 18–23 years old, COVID-19 (at least once), contact with COVID-19 patients, and the complete vaccination scheme. For IgG anti-N, we find similar associations except direct contact with COVID-19 patients (Table 3).

**Table 3:** Bivariate analysis of the risk factors with the presence of IgG Anti-S and Anti-N antibodies

Variable	Category	IgG Anti-S		naOR	95%CI	*P* value	IgG Anti-N		naOR	95%CI	*P* value
		Yes	No				Yes	No			
**Age**	18–23 years old	734	594	**2.24**	**1.45–3.46**	**< 0.001**	549	779	**2.05**	**1.27–3.31**	**< 0.001**
	24–29 years old	32	58				23	67			
**Gender**	Female	508	444	0.92	0.74–1.15	0.48	384	568	1.0	0.80–1.25	1
	Male	258	208				188	278			
**Comorbility**	Yes	240	193	1.09	0.86–1.36	0.48	176	257	1.02	0.81–1.28	0.89
	No	526	459				396	589			
**Vaccination**	Yes	759	640	2.37	0.91–6.18	0.08	567	832	2.39	0.81–7.05	0.12
	No	6	12				4	14			
**COVID-19 infection**	Yes	369	273	**1.29**	**1.05–1.59**	**0.019**	280	362	**1.28**	**1.04–1.59**	**0.023**
	No	397	379				292	484			
**Contact with** **COVID-19 patient**	Yes	503	384	**1.33**	**1.08–1.66**	**0.009**	371	516	1.18	0.95–1.47	0.14
	No	263	268				201	330			
**Complete vaccination scheme**	Yes	588	438	**1.62**	**1.28–2.07**	**< 0.001**	455	571	**1.90**	**1.48–2.44**	**< 0.01**
	No	172	207				112	267			

naOR: Unadjusted Odds Ratio; 95%CI: Miettinen Confidence interval 95%

Finally, the multivariate test was refined and used to confirm that the age between 18 and 23 years (ORa 2.39), contact with COVID-19 patients (ORa 1.35), and complete vaccination scheme (ORa 1.58) were associated with the presence of IgG anti-S. Whereas the age between 18 and 23 years (ORa 2.08) and the complete vaccination scheme (ORa 1.90) were related to the presence of IgG anti-N in the analyzed population (Table 4).

**Table 4:** Adjusted multivariate analysis of the risk factors with the presence of IgG Anti-S and Anti-N antibodies in the analyzed population

Variable	IgG Anti-S			IgG Anti-*N*		
	ORna^1^	ORa^2^	95%CI^3^	ORna^1^	ORa^2^	95%CI^3^
**Age 18–23 years old**	2.25	2.39	1.54–3.72	2.06	2.08	1.27–3.40
**Contact with COVID-19 patients**	1.34	1.35	1.09–1.69	----	----	----
**Complete vaccination scheme**	1.62	1.58	1.24-2.00	1.90	1.90	1.48–2.45

^1^ Unadjusted Odds Ratio. ^2^ Adjusted OR. ^3^ 95%CI: Miettinen 95% confidence interval

## Discussion

Since the first case of COVID-19, a total of 770, 778, 396 cases and 6,958,499 deaths have been reported until September of 2023 [34]. This disease is associated with the infection by SARS-CoV-2 and has symptoms such as cough, fever, myalgia, fatigue, asthenia, and headache [35]. All populations are sensitive to the infection by SARS-CoV-2 [36]; however, during the first months of the pandemic, higher incidences were reported in third age patients; later, several studies provided information about a higher prevalence in the younger population during 2020 [37]. Seroprevalence studies in youth populations provide information about the number of asymptomatic cases [38] or estimations about the course of the disease [39] in those who live and attend crowded places such as universities in which they can acquire and disseminate the virus [40, 41].

In this study, we aimed to analyze the prevalence of IgG antibodies against the S and N proteins of SARS-CoV-2 in eighteen centers of the Autonomous University of Guerrero. Patients had between 18 and 29 years old, and around 36.9% reported having at least one comorbidity as obesity (23.1%), asthma (6.3%), EPOC (1.7%), hypertension (1%), diabetics (0.6%), rheumatoid arthritis (0.2%), cancer (0.2%), and lupus (0.1%). These comorbidities predispose to develop severe disease or hospitalization [42, 43]. Hypertension, diabetes, and obesity are predictors of worst prognostic due to the endothelial damage of patients, oxidative process, and cellular/tissue inflammation. Also, obesity and asthma generate hypoventilation states that are considered bad prognostic in COVID-19 [44], while autoimmune diseases, such as arthritis and lupus, generate immunosuppression states and promote susceptibility to infections by microorganisms as SARS-CoV-2 [45, 46].

Vaccination against COVID-19 is safe and necessary to prevent the infection by SARS-CoV-2. In our population, 98.7% applied at least one dose of the vaccines Pfizer, Sinovac, AstraZeneca, and CanSino. Those vaccines are based on mRNA, complete virus, and viral vectors, respectively. Previous studies reported that antibody levels after applying mRNA vaccines are higher than those generated by natural infection. In contrast, antibody levels generated by the application of viral vector are equal to those generated by natural infection [47, 48]. Also, it has been reported that the aforementioned vaccines promote cellular and humoral immune response, associated with the production of IgA, IgM, and IgG, and the development of memory T and B cells against epitopes derived from S protein or the RBD of SARS-CoV-2 [49, 50]. It has been reported that heterologous boost induces a higher concentration of neutralizing antibodies in comparison to a homologous boost [51, 52]. In this study, most of the patients received heterologous doses, and the most administered vaccine was AstraZeneca (42%), which has been proven to increase the humoral immune response [53].

Seroprevalence studies evaluate the number of patients that were positive for the infection or vaccinated against the SARS-CoV-2 [54]. Antibody detection generates knowledge about past infection, transmissibility, response, and efficacy of vaccination [15] to establish health politicizes and epidemiological control [19]. In this study, we reported a total seroprevalence of 54.5%, in which, 14.1% corresponds precisely to IgG anti-S protein. Differences between the seroprevalence of both proteins could be due to S-protein being the main target antigen used to design and develop vaccines [9, 55]. Whereas antibodies against the N-protein are related to the early phases of the infection [11]. Similar studies performed on students have reported contrasting results. In the U.S.A. during 2020, the seroprevalence was 4.0-4.7% [24–26], while during 2021, the reported seroprevalence was from 22.5 to 46.8% [27, 28]. Antibody levels vary according to region and time [54]. We observed changes in the seroprevalence according to the geographic regions of the state; the highest rate was reported in Acapulco, Centro, and Costa Grande. These differences could be attributed to factors such as: cultural practices, mitigation efforts, health infrastructure, political decisions for the prevention and control of COVID-19, and the population flow of each region [56, 57]. Previous studies reported a diminution of the incidence of COVID-19 in zones with trim or without universities, compared with the prevalence reported in universities with high enrollment, due to constant socialization and behaviors promoting development of the disease [58]. Reported seroprevalences are due to the high grade of immunization in our population; this could affect the infectious rate and have a positive effect on the diminution of the number of infected patients, which is commonly denominated as community immunity [59, 60].

Finally, we associated analyzed factors with the production of antibodies. We reported a positive relation between age, COVID-19, contact with a COVID-19 patient, and vaccination with IgG against S and N proteins of SARS-CoV-2. Age is directly related to a progressive reduction in the ability of the immune system to trigger effective cellular and humoral responses against the infection or vaccines [61]. While COVID-19 and contact with COVID-19 patients promote antibody production through direct contact with the virus and the induction of innate and adaptive immune response that confers long-term protection; particularly, antibodies can be detected days after the start of the symptoms and are detectable several months after the infection [62]. Vaccines induce the production of IgM, IgA, and IgG during the first twelve days; these antibodies can neutralize and reduce the transmission of the disease [63, 64]. After vaccine application, the kinetics of antibody production is around three weeks and declines after that time [65, 66], and cellular immune response declines around four and fourteen weeks after the boost in mRNA vaccines [67]. In this study, the participants had an elapsed time of around six months since the last boost, which could explain the negative patients to IgG antibodies in vaccinated students.

Further investigations need to be performed in order to analyze the presence of IgG antibodies against the other structural proteins of SAR-CoV-2 (E and M) or evaluate the neutralizing capacity of IgG antibodies in order to have complete epidemiological data about the vaccination and natural infection by SARS-CoV-2 in university students. However, our results provide evidence of the high seroprevalence of anti-S and anti-N antibodies, which can promote collective immunity in this population.

## Conclusion

We reported a seroprevalence of 54.5% against the S and N proteins. The highest seroprevalence of IgG antibodies was reported in Acapulco and Costa Chica, which could be due to the high population rate and cultural resistance to safety measures against COVID-19. Also, this seroprevalence reflects the high vaccination coverage and natural infection by SARS-CoV-2. Evaluating IgG antibodies against the S and N proteins is fundamental to understanding the impact and deceleration of the pandemic in university students in Guerrero.

### Electronic supplementary material

Below is the link to the electronic supplementary material.

Supplementary Material 1

Supplementary Material 2

Supplementary Material 3

## Data Availability

The datasets used and/or analyzed during the current study are available from the corresponding author on reasonable request.

## Abbreviations

COVID-19: Coronavirus disease 2019
SARS-CoV-2: Severe acute respiratory syndrome-coronavirus-2
RBD: Receptor binding domain
ACE2: Angiotensin-converting enzyme 2
+ssRNA: Positive-sense single-stranded RNA virus
ORF: Open reading frames; nsp: non-structural proteins
RNP: Ribonucleoprotein complex
ELISA: Enzyme-linked immunosorbent assay
MAP8: Multiantigenic peptides of eight ramifications
