# Supplementary material for: Detection of IgG antibodies against the receptor binding domain of the spike protein and nucleocapsid of SARS-CoV-2 at university students from Southern Mexico: a cross-sectional study
Source: BMC Infect Dis. 2024 Jun 12;24:584. doi: 10.1186/s12879-024-09435-5 (PMC11170790; doi:10.1186/s12879-024-09435-5)
Supplement: Supplementary file 1 — Supplementary Material 1 [file 12879_2024_9435_MOESM1_ESM.docx]

**Supplementary material 2.- Geographic location of all the educative centers included in the study.**

| Educative center | Sample (number) | Municipality | Region | Geographical coordinate |
| --- | --- | --- | --- | --- |
| Facultad de Contaduría y Administración | 554 | Acapulco de Juárez | Acapulco | 16.874848615327185”N, 99.8850272344185”W |
| Facultad de Medicina |  | Acapulco de Juárez |  | 16.872637118868322”N, 99.88615727004648”W |
| Facultad de Enfermería no.2 |  | Acapulco de Juárez |  | 16.874019322995856”N,  99.88896433261783”W |
| Escuela Superior de Psicología |  | Acapulco de Juárez |  | 16.87568845214916”N, 99.88012489028814”W |
| Facultad de Odontología |  | Acapulco de Juárez |  | 16.87414602042611”N, 99.88776776008463”W |
| Facultad de Derecho Acapulco |  | Acapulco de Juárez |  | 16.874089689072683”N, 99.88955157679365”W |
| Facultad de Ciencias Químico Biológicas | 368 | Chilpancingo de los Bravo | Centro | 17.53635273680621”N, 99.49652907677718”W |
| Facultad de Ingeniería |  | Chilpancingo de los Bravo |  | 17.536691320659934”N, 99.49525235813331”W |
| Facultad de Derecho Chilpancingo |  | Chilpancingo de los Bravo |  | 17.537497797889575”N, 99.49687164794207”W |
| Escuela Superior de Enfermería no.1 |  | Chilpancingo de los Bravo |  | 17.556263340155624”N, 99.50473280561191”W |
| Escuela Superior de Ciencias Naturales |  | Chilpancingo de los Bravo |  | 17.485595344499647”N, 99.46446879027303”W |
| Escuela Superior de Enfermería no.3 | 177 | Ometepec | Costa Chica | 16.685133280870748”N, 98.40420383446855”W |
| Facultad de Medicina Veterinaria y Zootecnia no. 2 |  | Cuajinicuilapa |  | 16.48035530092768”N, 98.430156790079”W |
| Facultad de Medicina Veterinaria y Zootecnia no.3 | 52 | Tecpán de Galeana | Costa Grande | 17.235171678169966”N, 100.63074936144417”W |
| Facultad de Ciencias Agropecuarias y Ambientales | 137 | Iguala de la Independencia | Norte | 18.355403210164138”N, 99.54907881418471”W |
| Escuela Superior de Enfermería no.4 |  | Taxco de Alarcón |  | 18.550814152321895”N, 99.60164696461234”W |
| Escuela Superior de Enfermería no.5 | 130 | Coyuca de Catalán | Tierra Caliente | 18.3178831395214”N, 100.6939207314125”W |
| Facultad de Medicina Veterinaria y Zootecnia no.1 |  | Ciudad Altamirano |  | 18.341052726776454”N, 100.64825424976732”W |
