# Supplementary material for: Detection of IgG antibodies against the receptor binding domain of the spike protein and nucleocapsid of SARS-CoV-2 at university students from Southern Mexico: a cross-sectional study
Source: BMC Infect Dis. 2024 Jun 12;24:584. doi: 10.1186/s12879-024-09435-5 (PMC11170790; doi:10.1186/s12879-024-09435-5)
Supplement: Supplementary file 2 — Supplementary Material 2 [file 12879_2024_9435_MOESM2_ESM.docx]

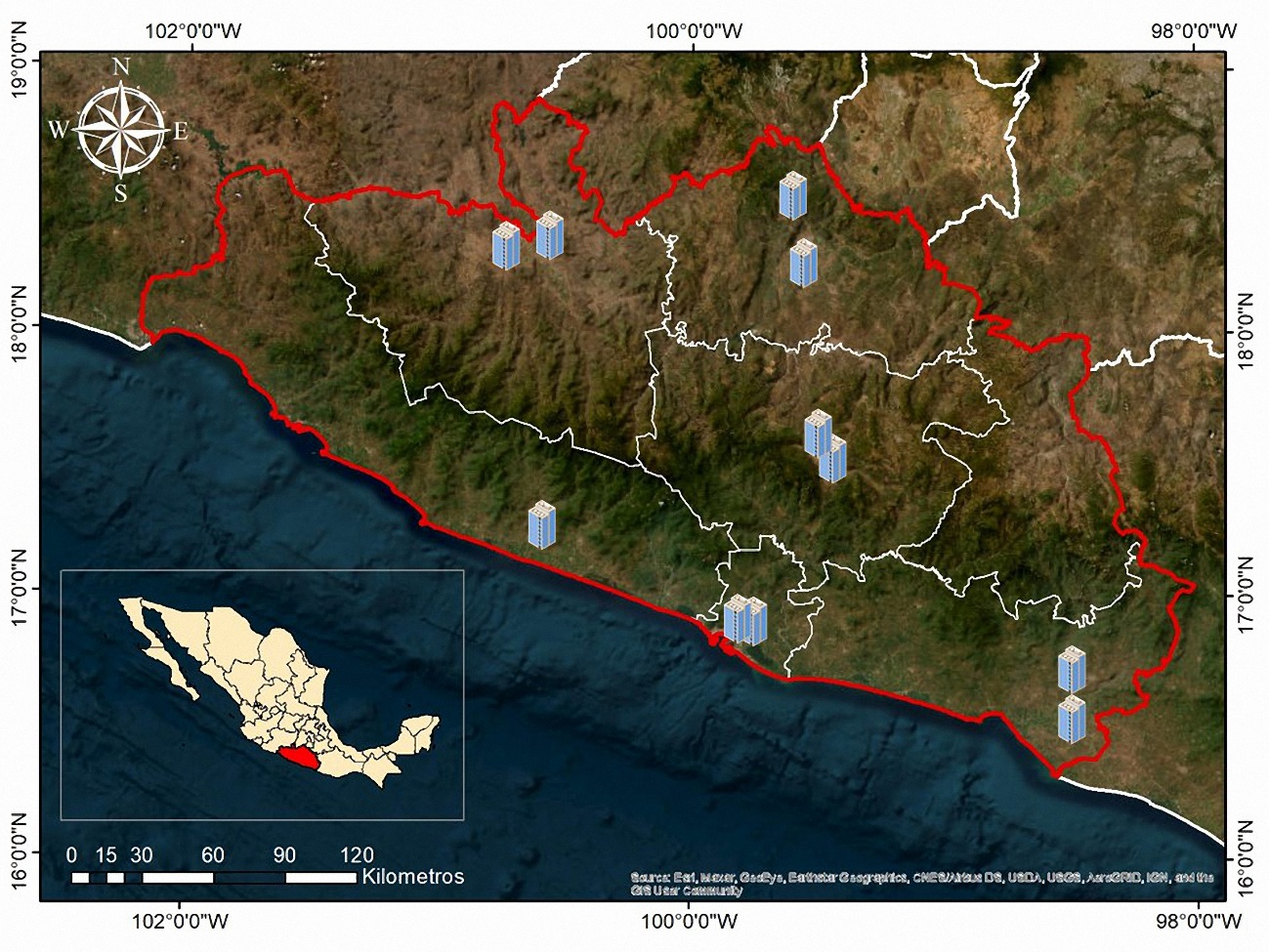


**Supplementary material 2.- Territorial map of the state of Guerrero in Mexico.** The map included the eighteen educational centers included in this study. Buildings are schematic representations of the localization of each center. Red and white lines are used to delimit the state of Guerrero and separate the state's seven regions.
