# Supplementary material for: Detection of IgG antibodies against the receptor binding domain of the spike protein and nucleocapsid of SARS-CoV-2 at university students from Southern Mexico: a cross-sectional study
Source: BMC Infect Dis. 2024 Jun 12;24:584. doi: 10.1186/s12879-024-09435-5 (PMC11170790; doi:10.1186/s12879-024-09435-5)
Supplement: Supplementary file 3 — Supplementary Material 3 [file 12879_2024_9435_MOESM3_ESM.docx]

| **Vaccine** | **Categories** | **% (n)** |
| --- | --- | --- |
| **Vaccine second dose** | AstraZeneca | 27.9 (395/1418) |
|  | SinoVac | 23.3 (331/1418) |
|  | CanSino | 1.3 (18/1418) |
|  | Pfizer/BioNTech | 21.3 (302/1418) |
|  | Others* | 1.0 (15/1418) |
| **Vaccine boost dose** | AstraZeneca | 40.0 (596/1418) |
|  | SinoVac | 6.8 (96/1418) |
|  | CanSino | 8.1 (115/1418) |
|  | Pfizer/BioNTech | 8.3 (117/1418) |
|  | Others* | 1.8 (25/1418) |

**Supplementary material 3.- Applied vaccines in the studied population**

***** Sputnik V, Moderna and Johnson & Johnson
